# Pathophysiology and neurologic sequelae of cerebral malaria

**DOI:** 10.1186/s12936-020-03336-z

**Published:** 2020-07-23

**Authors:** Nicoline Schiess, Andres Villabona-Rueda, Karissa E. Cottier, Katherine Huether, James Chipeta, Monique F. Stins

**Affiliations:** 1 Department of Neurology, Johns Hopkins School of Medicine, 600 N. Wolfe St., Meyer 6-113, Baltimore, MD 21287 USA; 2 Malaria Research Institute, Dept Molecular Microbiology Immunology, Johns Hopkins School of Public Health, 615 N Wolfe Street, Baltimore, MD 21205 USA; 3 Department of Paediatrics, University Teaching Hospital, Nationalist Road, Lusaka, Zambia; 4 Present Address: BioIVT, 1450 South Rolling Road, Baltimore, MD USA; 5 Johns Hopkins School of Medicine, Baltimore, MD 21287 USA

**Keywords:** Cerebral malaria, Neurologic sequelae, Blood brain barrier, Inflammation, Heterogeneity

## Abstract

Cerebral malaria (CM), results from *Plasmodium falciparum* infection, and has a high mortality rate. CM survivors can retain life-long post CM sequelae, including seizures and neurocognitive deficits profoundly affecting their quality of life. As the *Plasmodium* parasite does not enter the brain, but resides inside erythrocytes and are confined to the lumen of the brain’s vasculature, the neuropathogenesis leading to these neurologic sequelae is unclear and under-investigated. Interestingly, postmortem CM pathology differs in brain regions, such as the appearance of haemorragic punctae in white *versus* gray matter. Various host and parasite factors contribute to the risk of CM, including exposure at a young age, parasite- and host-related genetics, parasite sequestration and the extent of host inflammatory responses. Thus far, several proposed adjunctive treatments have not been successful in the treatment of CM but are highly needed. The region-specific CM neuro-pathogenesis leading to neurologic sequelae is intriguing, but not sufficiently addressed in research. More attention to this may lead to the development of effective adjunctive treatments to address CM neurologic sequelae.

## Background

Malaria is transmitted through the bite of *Plasmodium*-infected female *Anopheles* mosquitoes. It remains one of the most common vector-transmitted diseases, leading to a high disease morbidity and mortality. Although there are several *Plasmodium* species with the potential to cause disease, *Plasmodium falciparum* and *Plasmodium vivax* are the main two species responsible for most complications in humans, with *P. vivax* more prevalent in South East Asian countries, and India [1–3]. In 2018, there were roughly 228 million cases of malaria world-wide resulting in 405,000 deaths [1]. Of these deaths, 67% (272,000) were in children under the age of 5 years [1].

Multiple complications can occur as a result of *P. falciparum* infection, with cerebral malaria (CM) causing some of the highest mortality rates [1, 4, 5]. Furthermore, patients that survive CM can remain with life-long post CM sequelae, especially neurological deficits, affecting quality of life [6]. Severe malaria, due to *P. falciparum* infection, presents differently in children than adults, especially regarding the onset of CM. Whereas paediatric CM mortality is reportedly lower than adult CM mortality, paediatric CM is associated with a higher rate of seizures and post-CM neurocognitive deficits [7, 8]. These variances in CM disease presentation may arise due to differences in the immature brain, including differences in host responses of the cerebral vasculature in different brain regions to sequestration and the magnitude of inflammation. This review focuses on the underlying immunopathophysiological mechanisms of paediatric *P. falciparum* malaria and subsequent neurological sequelae as seen in sub-Saharan Africa.

### Host genetic susceptibility and resistance

Given that more than one million children per year were dying from *P. falciparum* in Africa alone prior to the twenty-first century [4], malaria is, from a genetic standpoint, the evolutionary driver resulting in genetic erythrocyte diseases such as sickle-cell, thalassaemia and glucose-6-phosphate dehydrogenase deficiency. This is supported by the observations that, despite homozygote mortality, the HbS allele has a high prevalence in areas endemic with malaria as well as the observation that independent genetic mutations have developed in different ethnic and geographical populations [9]. Other host genetic factors contributing to CM susceptibility include inflammatory factors and regulatory regions, such as Type 1 Interferon receptor variants in Malawi [10], IL17 in Nigeria and IL4 and IL22 in populations in Mali [11, 12]. In addition, earlier reports showed a role for intercellular adhesion molecular -1 (ICAM-1) Kilifi variants in CM [13]. A recent study in Kilifi, Kenya, identified 15 genes associated with increased paediatric malaria [14], and an Indian study identified TNF polymorphisms [15]. In addition, epidemiological studies reported association of outcomes of malarial infections with age and previous exposure to epigenetic modifications [16–19]. This arises from a recent discovery that the production of the citric acid cycle metabolites succinate and fumarate increased during severe malaria, including CM. These metabolites can serve as modulators of epigenetic enzymes, such as histone and DNA demethylases [20]. There is growing evidence that recurrent parasite infections, by invoking hyper responsiveness of the Toll-like Receptors (TLR) ligand stimulation, can result in epigenetic modifications with phenotypes showing resistance to malaria [21]. Indeed, these epigenetic modifications were reported among *Plasmodium* infected Kenyan children [16]. Coinfections in paediatric CM patients, such as HIV, are considered independent risk factors for death. Autopsy studies have demonstrated a two-fold increase in intravascular monocytes and platelets in HIV infected children who died from CM [22]. In addition, increased T cell presence was observed in human CM brains with HIV co-infection [23, 24]. It is likely that in co-infected patients, the HIV associated immune dysregulation further amplifies the pathological damage of CM, leading to increased T cell influx into the brain [22, 24, 25]. Taken together, various host factors contribute to susceptibility to severe malaria and, although there are differences among regions, factors associated with strong host-immune responses appear key.

### Clinical characteristics

**Fig. 1:**
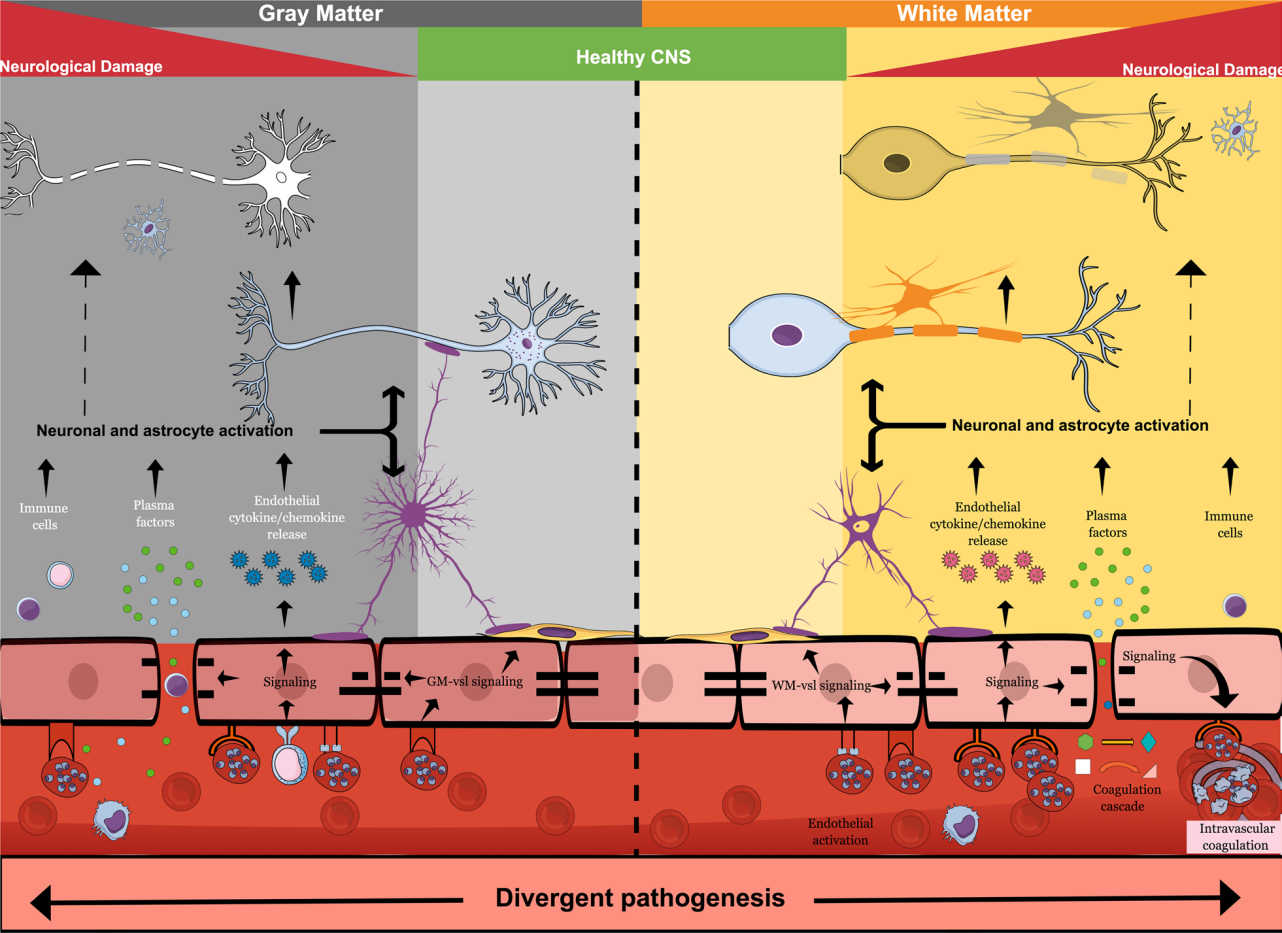
Graphical abstract of cerebral malaria pathogenesis. Cerebral malaria pathology manifests itself differently in white matter and gray matter of the brain. Whereas haemorragic punctae are abundant in white matter, they are not obvious in gray matter. The cerebral vasculature in these brain areas is different, which may lead to differential attachment of PRBC—as guided by *var* gene expression—of PfEMP1 and resulting activation of alternate signalling pathways in the brain endothelial vasculature in these regions. The release of chemokines and cytokines from the inflamed BBB endothelium towards the brain, in conjunction with the opening of the blood brain barrier that allows ingress of both neurotoxic plasma substances and soluble *Plasmodium* factors into the brain, leads to astroglial activation. This, together with an influx of immune cells, causes neurological damage that is responsible for the post CM neurologic sequelae

CM is the most severe neurological complication of the infection by *P. falciparum* and is a clinical syndrome whose hallmark is impaired consciousness, with coma being the most severe manifestation [26]. Clinical features of pediatric malaria, including CM, involve a relapsing diurnal fever, which is produced after parasite release upon rupture of *Plasmodium* infected red blood cells (PRBC), secondary to asexual replication and cytokine release [9, 27]. Patients with acute infection can present with a diffuse CM encephalopathy, a rapid progressive coma, and/or seizures without return to consciousness. In some cases, focal neurologic signs are present [28]. At the end stages of disease, children often display signs of brainstem dysfunction, such as abnormal pupillary and corneal reflexes, a dysconjugate gaze and irregular breathing patterns [28–32]. Although some sequelae, such as cortical blindness, improve with time, long-term clinical follow-up assessments in paediatric CM survivors showed elevated persistence of neurological sequelae, including hemiplegia, ataxia, paresis, seizure disorders, language deficits, altered behaviour, severe cerebral palsy and cognitive impairments [28, 29, 32, 33]. These neurologic sequelae may lead to an impaired quality of life and loss of disability adjusted life years. The exact underlying factors that play a role in the neuropathogenesis leading to poor neurological outcome in children are unclear. However, autopsy findings have ascertained that intravascular sequestration of *Plasmodium*-infected red blood cells is associated with perivascular damage, including axonal injury, myelin loss and breakdown of the blood brain barrier (BBB) [34], as reflected in Fig. 1. How exactly sequestration leads to BBB breakdown is unclear. As discussed later, sequestration together with soluble *Plasmodium* factors, may have both direct and indirect effects on BBB integrity, which may be amplified by the cytokine storm and influx of plasma factors, including albumin, that are toxic to neurons. Interestingly, in paediatric African CM patients, sequestration reportedly occurs in the brain vasculature irrespective of the region, however postmortem pathology revealed different host vascular responses [34, 35]. A predominance of multiple haemorrhagic punctate lesions is observed in white matter areas and corpus callosum, but not visible in other brain regions, such as gray matter or basal ganglia [35]. Moreover, adult *P. falciparum* CM also features a predominance of punctate white matter damage, as shown by postmortem pathology and magnetic resonance imaging (MRI) [36, 37]. Taken together, this suggests that potential phenotypic heterogeneity in the local host vasculature [38] that can attract differential PRBC sequestration can lead to alternate host responses [39] (Fig. 1).

The clinical World Health Organization (WHO) diagnostic criteria for CM, (*P. falciparum* on blood smear, coma and no other known cause of coma) [40], has the potential to result in misdiagnoses. Using these criteria, an autopsy study conducted in Malawi [35] reported that 23% of clinically diagnosed CM cases were, in fact, a different pathology entirely. This has the potential to skew the results of post CM cognitive studies in that some children might already have pre-existing neurocognitive problems. Fundoscopy and diagnosis of retinopathy has been shown to improve the specificity of the clinical diagnosis of CM, although the retinopathy appears to be less specific in adults [8, 41, 42]. CM-retinopathy is a constellation of ocular changes that includes retinal whitening, retinal haemorrhages, vascular changes and papilledema, and increased expression of vascular cell adhesion molecule-1 (VCAM-1) [41, 43, 44]. The severity of malaria retinopathy is also positively correlated with an increased risk of death [41, 43, 44]. Even after resolution of *Plasmodium* infection, neurologic symptoms persist in almost one-fourth of children with retinopathy-positive CM [28]. In addition, retinopathy negative CM patients were found to have pre-existing neurologic conditions, which subsequently allows for the possibility of inaccurate post CM neurological assessments [8]. Unfortunately, not all clinicians have access to funduscopes, as such instrumentation is relatively expensive for LIMC. Increased access to affordable funduscopy adaptations, e.g. by using adapted cell phones in combination with appropriate algorithms and training will improve appropriate diagnosis of CM and may provide predictions on risk of neurologic sequelae.

Access to other non-invasive imaging modalities, such as MRI, is extremely limited in low and middle income countries. Nevertheless, MRI studies have provided further insight into the pathogenesis and cause of death of sub-Saharan paediatric CM patients. MRI findings in 120 children with retinopathy-positive CM demonstrated increased cerebral volume (50%) and T2 brain abnormalities, suggesting inflammation in the basal ganglia (84.2%), white matter (71,7%), brain stem, thalamus (40%), corpus callosum (49,2%) and cerebellum (49,2%) ^30^. A subsequent 1.5 Tesla MRI study performed on 16 retinopathy-positive CM children in Zambia indicated that diffusion weighted imaging abnormalities spared the gray matter, indicating a likely vasogenic, as opposed to cytotoxic, oedema. Micro-haemorrhages and parasite sequestration occurred in the same white matter regions. The diffusion weighted imaging results are consistent with micro-haemorrhages and parasite sequestration co-occurring in white matter regions with vascular congestion [45]. These regional differences in MRI findings suggest potential differences in host vasculature between these white- and gray matter regions (Fig. 1).

MRI findings that have been associated with poor to fatal CM outcomes include signs of elevated intracranial pressure, cerebral oedema, decreased cerebrospinal fluid (CSF) volume posterior cerebral involvement, thalamic and supratentorial gray matter lesions and patchy areas of lobular involvement [31, 45]. MRI studies have also demonstrated a clear link between cerebral oedema, depth of coma, and increased mortality [30].

### Neuropathogenesis of cerebral malaria

CM pathogenesis is multifaceted and, until recently, has been complicated by disease heterogeneity, often inaccurate clinical case classifications and a lack of large, clinical prospective studies [35]. Although already adopted by some groups with access to funduscopy, an overall standard use of CM-retinopathy as an inclusion criterion for studies will likely result in more precise follow up for assessing the late sequelae of CM. The enigma of CM neuropathogenesis and resultant coma has confounded scientists for decades as the pathogen itself, residing inside PRBC’s, does not directly or physically enter the central nervous system (CNS) due to the BBB, but remains inside the vascular lumen (Fig. 1). Yet, severe neurological symptoms, including coma, are a hallmark in CM and pathological evidence of neuronal injury have also been demonstrated by elevated tau levels in the CSF of children with CM [46]. This highlights the vital role of the BBB endothelium in CM, as the BBB is at the interface of PRBC intravascular sequestration and underlying neuronal damage (Fig. 1).

Two main theories (1) the “mechanical hypothesis” and (2) the “cytokine storm” hypothesis provide an explanation underlying CM-neuropathogenesis. The *mechanical hypothesis* is based on the contribution of intravascular sequestration of PRBCs that results in multiple consequences, including vascular congestion, hypo-perfusion and localized hypoxia [47, 48]. In addition, differences in local blood flow may contribute to increased intracranial pressure in CM and lobular differences. This may be due to either differences in vascular supply, e.g. occipital lobe via posterior cerebral artery versus other lobes via the circle of Willis, due to PRBC sequestration or due to a combination of these factors. Together, these factors ultimately lead to a breakdown of the BBB, cerebral oedema and a pro-thrombotic state [49–51]. The parasite-encoded *P. falciparum* erythrocyte membrane protein-1 (PfEMP-1) is expressed on PRBC surfaces and interacts with host receptors. PfEMP-1 is devised to save PRBCs from clearance by the spleen and is responsible for intravascular PRBC sequestration. PfEMP-1 is encoded by a variable gene (var-gene) and, depending on which var-gene is expressed, interacts with various host adhesion receptors, such as ICAM-1, EPCR and CD36 [52–55]. Binding of PRBC expressing differential PfEMP-1, as encoded by the *var* gene family, to its respective receptor leads to downstream host signalling, including activation of inflammatory and coagulatory pathways, eventually leading to loss of BBB integrity and encephalopathy (Fig. 1). In addition, as assessed in in vitro experiments with brain endothelial cells, differential host endothelial responsiveness may affect development of CM in patients [56]. Post-mortem data, animal models for CM and in vitro data, demonstrate that PRBC sequestration correlates with brain vascular activation. This is shown by the presence of large vesicular nuclei, endothelial destruction, activation of transcription factor NF-κB, increased expression of cell adhesion molecules, such as ICAM-1, VCAM-1, E-selectin, cytokine release and BBB breakdown [35, 57–62]. Endothelial damage in CM is also demonstrated by changes in the endothelial glycocalix upon exposure to PRBC, both in vitro [63] and in vivo in human CM [64] and murine experimental CM (eCM) [65] and release of endothelial vesicles into the circulation [66].

Although several studies correlate the degree of PRBC sequestration in the brain to increased CM severity [34, 67, 68], the extent to which this correlates with clinical symptoms, coma development and mortality in CM is debated [69]. Therefore, as proposed by the “*cytokine storm hypothesis*”, peripheral inflammation, neutrophil activation [60] and increased circulations of multiple serum cytokines such as TNF, IFNγ, and IL-2, IL-6, IL-8, and IL-10 contribute to the CM pathogenesis [12, 70]. When compared with patients with uncomplicated malaria, circulating IL-6, MCP-1 and vascular endothelial expression of CD61 are upregulated [56]. The increase in inflammatory markers are indicative of both immune cell and endothelial inflammation and associated with PRBC sequestration [57, 61]. Increased levels of soluble plasma neutrophil proteins and impaired neutrophil chemotaxis were found in retinopathy positive paediatric CM [60], indicative of neutrophil activation. These activated neutrophils may, similar to the intravascular localized monocytes [22], contribute to vascular activation. In addition, CD8 T cells have been found associated with the brain vasculature, both intravascular and perivascular and with the endothelial basal lamina where they can contribute to cerebral vascular activation, both in human and murine studies. Murine eCM studies indicated that those T cells that transmigrated further into the neuropil can damage neurons through release of Granzyme B and/or perforin [71, 72]. At this moment, it is not clear if the CD8+T cells preferentially invade specific white matter or gray matter brain regions. Additional mechanisms whereby neuronal damage occurs may involve caspases in select neurons, as shown in human CM [73].

Vascular integrity and lymphocyte transmigration can also be affected by sphingolipid alterations. For example, therapeutic sphingosine-1-phosphate (S1P) blocking agents, such as FTY720 decreased lymphocyte trafficking into the brain and lowered peripheral IFNγ levels [74]. Not all studies have found a relation between peripheral cytokines and cerebral oedema in CM [75].

Taken together, sequestration and inflammation, in conjunction with elevation of coagulation factors and alterations in blood metabolites, all contribute to CM neuropathogenesis [46, 70, 76–81], which may occur in a region-specific manner, e.g. white matter or gray matter. Regardless of either the cytokine storm- or sequestration-mediated CM hypothesis, the effects of endothelial activation can be seen incrementally even in subclinical presentations of parasitaemia as indicated by elevated serum levels of von Willebrand factor (VWF), soluble ICAM-1, and soluble-VCAM-1 [82, 83]. This signifies the paramount importance of the contribution of the BBB endothelium in the pathogenesis of CM and while the exact relationship between cerebral oedema and peripheral inflammation has yet to be fully elucidated, it is likely that they are also correlated with downstream neural stem cell repair processes.

### Post CM neuro-sequelae and potential mechanisms

Post CM, persistent neurologic sequelae, including cognitive impairment, motor skills, visual coordination, seizures and attention deficit hyperactivity disorder, occur in up to 25% of paediatric survivors [28, 33, 84–86]. The highest risk for deficits in motor, language, and social development was for children under 5 years of age [87]. Between 3 and 6 months post infection, cognitive deficits specific to working memory can intensify with language development being the most consistently affected in paediatric survivors [7, 32, 88, 89]. Cognitive impairments, including memory and attention, can persist for as long as nine years post CM episodes [84, 85]. Ten percent of paediatric CM survivors in one study had at least one mental health sequela with onset ranging from six to twelve months’ post infection and a median of 21 months follow up. The three top mental health disorders in this group were attention deficit hyperactivity disorder, conduct disorder and oppositional defiant disorder [90]. Post-CM, patients with increased externalizing behaviours (i.e. poor attention and aggression) were also reported [87, 91]. However, accurate assessment of neurologic sequelae can be challenging. Most studies done, thus far, on the long term sequelae of CM have used the 2000 clinical WHO definition of CM [7, 84, 85, 88, 91, 92]. This could mean that part of these children did not have true CM, or another underlying infectious condition as well. This opens the possibility that children were included who already had pre-existing neurocognitive conditions or that this was due to the co-infection and not CM. Regardless, the presence of neurologic sequelae in both retinopathy positive and negative children has been well documented [29]. The retinopathy negative children display a different clinical pattern and presentation. Although retinopathy positive patients display more abnormalities on MRI in a variety of different brain regions, the percentage of patients with neurologic sequelae is similar [30]. The lower mortality in the retinopathy negative patients may be due to the presence of potentially protective- co-infections, skewing the immune responses ^30,45,93^. Interestingly various brain regions appear to be differentially affected. Due to diverse study cohorts in the different countries and assessment methodologies, these studies cannot be directly compared for behavioural assessment. However, it is clear that, as a result of complicated *Plasmodium* infections, there is a degree of behavioural difficulties in many cases that satisfy the criteria for a mental health disorder diagnosis.

Seizures are common in children with CM and, as a long-term consequence, sustained seizure disorders, often refractory to at least one antiepileptic medication may develop, even months after a CM episode [28, 29, 33, 84, 85, 94–96]. The presence of seizures could contribute to development of other post CM neurological sequelae, including developmental delays [7, 28]. In particular, acute seizures during CM episodes was an indicator for future developmental disabilities raising the question of whether acute seizures might promote epileptogenesis and increased risk for chronic epilepsy [28, 89, 93].

Elucidation of the mechanisms underlying the development of behavioural disorders in CM is challenging, especially as complex human behaviours are difficult to recapitulate in animal models. There are several animal models that are used to study CM pathogenesis, but there are ethical concerns in using primate models [97, 98] and these are very expensive. The animal model that is mostly used to study CM is the *Plasmodium berghei* ANKA strain in a murine model, which recreates a number of features observed in human CM: including vascular inflammation, haemorrhagic punctae in parts of the brain and a set of neurologic sequelae that can be measured in behavioural tests [99–101]. Like any model system for a disease, the *P. berghei* ANKA model is not ideal and has received critiques. Critiques on the murine eCM model revolved around the absence of a PfEMP-1 analogue in the parasite, limited sequestration, the findings of effective therapeutics in the eCM models *versus* their limited success in human CM [102] and perceived differences in T cell involvement in murine eCM *versus* human CM. Although authors rightfully caution against blindly extrapolating murine model data, caution to their stance is warranted as addressed at several scientific meetings and in publications [103–107]. Importantly, although previous work failed to show the presence of CD8+T cells in human CM brains [34], recently and due to updated and highly sensitive methodologies, their presence was confirmed in human CM brains; these were both intravascular and perivascular, transmigrated into the neuropil and associated with the choroid plexus, an important entry point into the ventricles and cerebrospinal fluid [23–25, 108]. In addition, differences in the measured outcomes of a study may also contribute to, e.g. the survival or incidence of neurologic sequelae in survivors. It is, therefore, imperative to compare results obtained in animal models with human pathology. Due to ethical issues and regulatory guidelines, availability of pathological human CM specimens for CM research is highly limited. Improved patient participation/consent from family members and improved access to the wider CM research community could greatly benefit future CM patients. Moreover, other outcomes than just survival should be taken into account, such as reductions in neurologic sequelae when evaluating new adjunctive treatments for effectiveness.

Several studies using the murine *P. berghei* ANKA eCM model demonstrated an increase in anxiety-like behaviours following resolution of *P. berghei* ANKA infection [109]. Inflammatory signalling, such as increased brain-cortical TNF levels in conjunction with either increased IL-6 or IL-1β, and alterations in growth factor levels, e.g. brain derived neurotrophic factor and neuregulin, can contribute to the development of anxiety in eCM and behavioural sequelae [109–112]. Endothelin family peptide ET-1 (Pre-proendothelin) also plays an important role in inducing BBB damage and produces a CM-like picture in rodent models [113]. Selective endothelin receptor antagonists also improved outcome in cognitive decline and decreased brain hemorrhages in mice [113–115].

In response to increased inflammation in eCM, Darling et al. showed the relevance of the receptor tyrosine kinase EphA at the BBB endothelium [116]. Upregulation of EphA2 was shown to be required for the loss of BBB junction proteins both in eCM and in human brain microvascular endothelial cells and for infiltration of CD8+T cell into the brain in the eCM model.

Taken together, these studies suggest that CM episodes, both in humans and in animal models can lead to neurological deficits. The presence of specific neuronal repair processes after resolution of *Plasmodium* infection is implicated, although the nature and efficiency of these underlying mechanisms is unclear. The neuronal damage inflicted during *Plasmodium* infection could be related to the extent of the inflammatory responses, the amount of PRBC sequestration or, post-infection, to the efficiency of the neurological repair mechanisms. Very little is known about post CM repair processes. Given the extent of the neuronal damage, neuro-progenitors will be migrating in and will need to replace these damaged neurons. Especially due to the still highly inflammatory environment in the brain, this process can be skewed and contribute to the neuro-sequelae. More research into the underlying molecular pathogenic and repair mechanisms of CM is needed. This research should focus on several key areas, including ameliorating brain vascular inflammation, breach of barrier function and neuronal damage. In addition, a focus on the neuronal repair processes in CM is needed. This may lead to identification of targets for adjunctive therapies targeting the brain vasculature, neuroprotection and neuro repair. Study outcomes should focus not only on survival, but also target neurologic sequelae and behaviour.

### Quest for novel adjunctive therapies

Effective adjunctive neuro-protective therapies are currently not available. Numerous attempts to develop therapies targeting neurological sequelae have failed or have even increased the incidence of adverse sequelae, as reviewed by Varo et al. [117]. These attempts have mainly focused on efforts to decrease sequestration, inflammation, coagulation, and oxidative stress and have thus far proven ineffective in reducing mortality and preventing adverse neurological outcomes [118–122]. For example, treatment of CM patients with dexamethasone, commonly used in other neurological disorders to reduce inflammation and combat vasogenic oedema, actually resulted in longer coma and increased neurological complications [123]. Adjunctive therapies attempting to counteract brain oedema with the osmotic diuretic mannitol, known to lower intracranial pressure, showed no beneficial effect in CM patients [124]. Also targeting inflammation, monoclonal anti-TNF antibody therapy worsened CM outcome and increased the incidence of post-CM neurologic sequelae [125]. Although pharmacologic reduction of TNF levels using pentoxifylline, a phosphodiesterase inhibitor, showed slight improvements in survival for humans in some studies [126, 127], others showed no benefit on clinical outcomes [128, 129], or actually increased mortality when studied in children [130]. Interestingly, decreases in hippocampal TNF and IL-6 levels were observed in a murine eCM model with cannabidiol treatment, which also increased brain derived neurotrophic factor levels, promoted eCM survival and prevented post-CM anxiety-like behaviours [111]. The dual role of TNF in neurogenesis could be due to differential downstream signalling effects, dependent on which TNF-receptor is involved. While signalling through TNFR1 significantly hinders neurogenesis, activation of TNFR2 contributes to survival and proliferation of neural stem cells [131]. Neurogenesis, as driven by neural stem cells in the sub granular zone of the hippocampus, is important in learning and memory function [132, 133]. In the murine eCM model, increased expression of TNFR1 and, to a higher degree, TNFR2 is seen [134, 135]. Consequently, a full blockade of TNF signalling may hinder its neurogenic actions and, thus, lead to higher neurocognitive deficits. Targeting other cytokines might show promise for possible interventional strategies. A study examining the post mortem serum and CSF of children in Ghana with CM showed elevation of IP-10, IL-8, MIP-1β, PDGFbb, IL-1ra, Fas-L, sTNF-R1, and sTNF-R2 [136]. Neuregulin, a neurotrophic growth factor was shown to be protective of eCM and was postulated as an effective adjunctive therapy to reduce CNS tissue injury [112]. Administration of neuregulin-1 resulted in a 73% increased survival in eCM, as well as a decrease in systemic and CNS cytokines; TNF, IL-6, IL-1α and CXCL10 [137].

Further attempts to elucidate the role of other potential contributors to improving survival include targeting the complement system with a C5/C5a receptor blockade [138] and blood flow/vascular health using inhaled nitric oxide [139] Nitric oxide has a role in the pathogenesis of CM with low peripheral concentrations contributing to the pathology [140, 141]. Nitric oxide based adjunctive therapy is also effective in eCM through a variety of mechanisms, indicating that direct nitric oxide administration or through dietary supplementation of citrulline may be beneficial as adjunct therapy [142].

Other adjunctive therapies that target inflammation and immune responses and show promise in eCM studies include the peroxisome proliferator-activated receptor gamma agonist rosiglitazone, which improves both survival and neurocognitive outcome [143, 144]. The cholesterol lowering drug atorvastatin [145] also dampens inflammation and thereby reduces both brain endothelial damage and BBB opening [146]. Moreover, rosglitazone boosted neuroprotective pathways and, in human studies, showed promise for treatment of uncomplicated malaria [117], but its effects in human CM are, thus far, unclear. Erythropoietin, a hormone produced by the kidneys, has been considered for use in CM as also has antioxidant and anti-inflammatory components in animal models. Murine studies have demonstrated positive, neuroprotective results, either alone [147] or when paired with artesunate [148]. However, clinical studies demonstrated that high erythropoietin levels are associated with extended coma and increased mortality [149]. As the BBB inflammation and loss of integrity also plays a central role in CM, a potential therapeutic approach of blocking EphA2 to protect the BBB from breakdown was suggested in recent eCM studies [116].

It is clear from both animal and clinical studies that, although high levels of inflammation and brain oedema have been associated with CM mortality and neuro-sequelae, addressing this pharmacologically still presents significant challenges. Completely suppressing inflammation is detrimental, but ameliorating inflammation appears beneficial in CM survival outcome and may likely be neuroprotective as well.

Many of these studies on adjunctive therapies in mice have examined mortality outcomes and did not assess neurocognitive impairment. Thus, it is possible that neurocognitive effects may have been missed. These types of studies could have strengthened the argument for adjunctive therapy. Additional research is needed to elucidate the complex neuropathology leading to long-term neurological deficits, to identify biomarkers predictive of the severity of the neuro-sequelae and how this can be addressed clinically with adjunctive neuroprotective treatments. This requires testing additional outcomes beyond survival, both short and long term and to include a focus on reduction of neurologic sequelae.

### Concluding remarks

CM is a devastating disease with complex neuro-pathophysiology that can lead to a variety of neurologic sequelae affecting a person throughout life. The heterogeneity of clinical symptoms and outcomes range from full recovery, to various neurologic sequelae and, often, to death. With improvements in healthcare in sub-Saharan Africa more paediatric CM patients are expected to survive. This population may develop lasting neurologic sequelae, thus contributing to a growing global health concern. Several clinical studies have identified specific characteristics of these neurological sequelae, including cognitive and, behavioural deficits and seizure disorders. Human post-mortem and imaging studies have increased understanding of the neuropathology. Murine eCM studies can address mechanisms involved in eCM development and genesis of neurologic sequelae. Although current research suggests a significant role for inflammation in eliciting neuronal damage and the development of post CM sequelae, more research is needed to address the more specific underlying molecular pathophysiological drivers and signalling mechanisms. This underlines the importance of collaboration among different fields, including clinical research, animal research, and exchange of samples as key to advancing existing knowledge of CM neuropathogenesis. Together, this may lead to identification of novel targets for adjunctive treatments to ameliorate post-CM neuro-sequelae.

## Data Availability

Not applicable.

## Abbreviations

BBB: Blood brain barrier
CM: Cerebral malaria
CSF: Cerebrospinal fluid
eCM: Experimental cerebral malaria
ICAM-1: Intercellular cell adhesion molecule-1
MRI: Magnetic resonance imaging
PRBC: *Plasmodium* infected red blood cells
PfEMP-1: *Plasmodium falciparum* erythrocyte membrane protein-1
PRBC: Plasmodium infected red blood cells
VCAM-1: Vascular cell adhesion molecule-1
WHO: World Health Organization
